# Effect of conditioned media from *Aeromonas caviae* on the transcriptomic changes of the porcine isolates of *Pasteurella multocida*

**DOI:** 10.1186/s12866-022-02683-y

**Published:** 2022-11-11

**Authors:** Nonzee Hanchanachai, Pramote Chumnanpuen, Teerasak E-kobon

**Affiliations:** 1 Interdisciplinary Graduate Program in Bioscience, Faculty of Science, Kasetsart University, 10900 Bangkok, Thailand; 2 Omics Center for Agriculture, Bioresources, Food, and Health, Kasetsart University (OmiKU), 10900 Bangkok, Thailand; 3 Department of Zoology, Faculty of Science, Kasetsart University, 10900 Bangkok, Thailand; 4 Department of Genetics, Faculty of Science, Kasetsart University, 10900 Bangkok, Thailand

**Keywords:** Gene expression, Porcine respiratory bacteria, Bacteria-bacteria interaction, Bacterial co-culture, Bioinformatics

## Abstract

**Background:**

*Pasteurella multocida* is an opportunistic pathogen causing porcine respiratory diseases by co-infections with other bacterial and viral pathogens. Various bacterial genera isolated from porcine respiratory tracts were shown to inhibit the growth of the porcine isolates of *P. multocida*. However, molecular mechanisms during the interaction between *P. multocida* and these commensal bacteria had not been examined.

**Methods:**

This study aimed to investigate the interaction between two porcine isolates of *P. multocida* (PM2 for type D and PM7 for type A) with *Aeromonas caviae* selected from the previously published work by co-culturing *P. multocida* in the conditioned media prepared from *A. caviae* growth and examining transcriptomic changes using RNA sequencing and bioinformatics analysis.

**Results:**

In total, 629 differentially expressed genes were observed in the isolate with capsular type D, while 110 genes were significantly shown in type A. High expression of genes required for energy metabolisms, nutrient uptakes, and quorum sensing were keys to the growth and adaptation to the conditioned media, together with the decreased expression of those in the unurgent pathways, including translation and antibacterial resistance.

**Conclusion:**

This transcriptomic analysis also displayed the distinct capability of the two isolates of *P. multocida* and the preference of the capsular type A isolate in response to the tough environment of the *A. caviae* conditioned media. Therefore, controlling the environmental sensing and nutrient acquisition mechanisms of *P. multocida* would possibly prevent the overpopulation of these bacteria and reduce the chance of becoming opportunistic pathogens.

## Background

*Pasteurella multocida* is a Gram-negative bacterium that could be isolated from respiratory tracts of both healthy and diseased animals [1–4]. It is an opportunistic pathogen that causes economically significant respiratory diseases in pigs, including pneumonic pasteurellosis, atrophic rhinitis, and porcine respiratory disease complex (PRDC) [2, 5–7]. *P. multocida* is classified into five capsular serotypes (A, B, D, E, and F) [8]. The capsular serotypes A and D are commonly isolated from both diseased and healthy porcine respiratory tracts [9–12]. The porcine strains of *P. multocida* frequently co-infect with other bacterial and viral pathogens [13, 14]; for example, the co-infection of *P. multocida* with *Mycoplasma hyopneumoniae* revealed more severe pneumonic lung-damaged areas compared to the single infection [15]. This bacterium can also survive in soil and water [16] and is considered a member of the porcine respiratory tract microbiome [16–24]. To discriminate between the pathogenic and commensal stages of *P. multocida*, virulence factors were widely studied, i.e., capsules, lipopolysaccharides, toxins, and outer membrane proteins, leading to the development of vaccines and diagnostic methods [25]. The microbial community of the porcine respiratory tracts could potentially impact the stage of *P. multocida.* Several reports used 16s rRNA and shotgun metagenomic sequencing methods to reveal the microbiota complexity of the porcine lungs, which consisted of multiple bacterial families (*Mycoplasmataceae*, *Flavobacteriaceae*, and *Pasteurellaceae*) and genera (*Acidovorax*, *Acinetobacter*, *Aeromonas*, *Enterobacter*, *Escherichia*, *Hafnia*, *Klebsiella*, *Macrococcus*, *Pasteurella*, *Proteus*, *Providencia*, *Shewanella*, *Shigella*, and *Weeksella*) [17, 19, 21–23, 26–28]. These microbiotas are influenced by daycare attendance, antibiotic treatment, and feeding routine [29, 30]. The commensal microbiotas provide a favourable effect on their host by outnumbering and competing with the pathogens [31]. Depending on environmental stimulation and interaction with other members, the microbiota members in the porcine respiratory tracts could transform from commensal bacteria to become primary or opportunistic pathogens. Previous reports could support the assumption that the same bacterial species were either commensal or pathogenic bacteria in the pig respiratory tracts, i.e., *Mycoplasma flocculare*, *Mycoplasma hyorhinis*, *Haemophillus parasuis*, *P. multocida*, and *Staphylococcus suis* [22, 31, 32].

A previous co-culture assay by Hanchanachai et al. studied interactions within the conditioned media prepared from porcine respiratory bacteria and *P. multocida* isolates of capsular serotypes A and D and found that the negative interaction was predominant in this bacterial community [28]. In contrast, most bacterial genera could inhibit the growth of *P. multocida* [28]. Several studies suggested that the interaction between members in the microbiome could promote host wellness by enhancing harvest, storage, energy metabolism [33, 34], and immune regulation [35, 36]. One common approach for examining these molecular mechanisms of the bacteria-host interactions was the high-throughput gene expression assay or transcriptomics by RNA sequencing (RNASeq) [37, 38]. For example, on the host response, the transcriptomic analysis of *P. multocida*-infected murine lungs found the upregulated genes related to immune responses, i.e., pattern recognition receptors (PRRs), chemokines, and inflammatory cytokines in the *Il*- and IFN-families [39]. For the pathogen response, Cheng et al. compared the expression of genes related to pathogenicity and virulence (including *fimA*, *tbpA*, *exbD*, *fur*, *oma87*, *pmHAS*, *nanH*, and *tonB*) of the high and low virulence strains of *P. multocida* in lung tissues. They found seven genes that showed increased expression in the high virulence strain in vitro and in vivo [40]. Another transcriptomic study of *P. multocida* during growth within the chicken hosts found the upregulated genes involved in amino acid transport and metabolism, energy production and conversion, e.g., *asnA*, *aspC*, *dcaA*, *dppA*, *gdhA*, *gltA*, *ilvH*, *napA*, *napC*, *napF*, *PM0092*, *PM0287*, *PM1460*, *ppc*, *ptfA*, and *purH* [41]. The growth of *P. multocida* in limited nutrient media also showed differential expression of genes related to amino acid biosynthesis and transport systems, outer membrane proteins, and heat shock proteins (*argA*, *argD*, *artP*, *artQ*, *argR*, *artl*, *asnA*, *brnQ*, *carA*, *crr*, *dsbA*, *dppA*, *dod*, *errf*, *greB*, *gpt*, *gntR*, *glpX*, *hsf*, *hofB*, *ilvC*, *memE*, *memB*, *metC*, *mtr*, *ompW*, *pcnB*, *purF*, *pyrD*, *rpL23*, *recA*, *rsgA*, *rpoH*, *rpL1*, *rpL3*, *rpS8*, *rpL10*, *rpL5*, *rpL21*, *rpL9*, *spa*, *tyrA*, *trpE*, *tig*, *tsf*, *tyrP*, *trpC*, *trpA*, *truA*, *trpD*, *trx*, *tolQ*, and *ttrB*) [42] while *P. multocida* growth in iron limitation increased expression of genes related to iron binding and transport (*exbBD*, *fbpABC*, *fecBCD*, *tonV*, and *yfeABCD*). It decreased the expression of genes involved in energy metabolism and electron transport (*eno*, *pgk*, and *gapdh*) [43]. During the in vitro growth, genes involved in protein transport, RNA degradation, amino acid metabolism, and nucleotide metabolism were highly expressed compared to the in vivo study [38]. These transcriptomic profiles provided molecular snapshots of how *P. multocida* responded to the in vivo and in vitro environments.

Despite several transcriptomic studies of *P. multocida*, the gene expression analysis of *P. multocida* interaction with other commensal bacteria has been limited. Our previous study revealed that multiple bacterial isolates from the porcine respiratory tracts could inhibit the growth of *P. multocida*, including *Aeromonas* sp., *Macrococcus* sp., and *Providencia* sp. [28]. Comparison of proteome profiles of *P. multocida* capsular serotypes A and D cultured in the conditioned media from *Aeromonas* sp., *Macrococcus* sp., and *Providencia* sp. revealed that the growth in the *Aeromonas* sp. conditioned media showed the most different proteome changes (unpublished results). Therefore, this study aimed to examine the transcriptomic profiles of the porcine isolates (capsular serotypes A and D) of *P. multocida* co-cultured in the conditioned media of *A. caviae*, a porcine respiratory bacterium, compared with their growth in the complete medium, using RNA sequencing and bioinformatic analysis. This study could explain molecular mechanisms involved in the interaction between *P. multocida* and *A. caviae*. The differentially expressed genes helped developing new methods to control or maintain the commensal stage of *P. multocida*.

## Results

### Growth of *P. multocida* isolates in the complete and *A. caviae* conditioned media

The growth of *P. multocida* isolates PM2 and PM7 was different in the complete and conditioned media. These isolates were inhibited entirely in the conditioned media prepared from *A. caviae*. For the growth in BHIB after 18 h, *P. multocida* isolates PM2 reached the maximum OD_600_ of 0.81 ± 0.08 while the isolate PM7 slower approached the maximum OD_600_ of 0.694 ± 0.05. The optical density values were lower for the growth in the conditioned media. The OD_600_ values of the undiluted or 100% conditioned media were very low (0 to 0.05 for PM2 and 0 to 0.04 for PM7). The ODs increased when growing the isolates in the diluted conditioned media at 10%, 20%, and 50% concentrations (≥ 0.4), except the isolate PM7 growth in the 50% conditioned media remained low (0.1) after 17 h. After calculating the IC_10_, IC_20_, and IC_50_ values, the IC_20_ values still inhibited the growth of *P. multocida*. They provided enough cell materials for the RNA extraction, whereas the IC_10_ concentration gave a similar change to the complete media, and the IC_50_ yielded dramatically limited bacterial cells. The chosen IC_20_ concentrations for the isolates PM2 and PM7 were 50% and 10% v/v of the *A. caviae* conditioned media, respectively.

### Transcriptomic data analysis

**Fig. 1:**
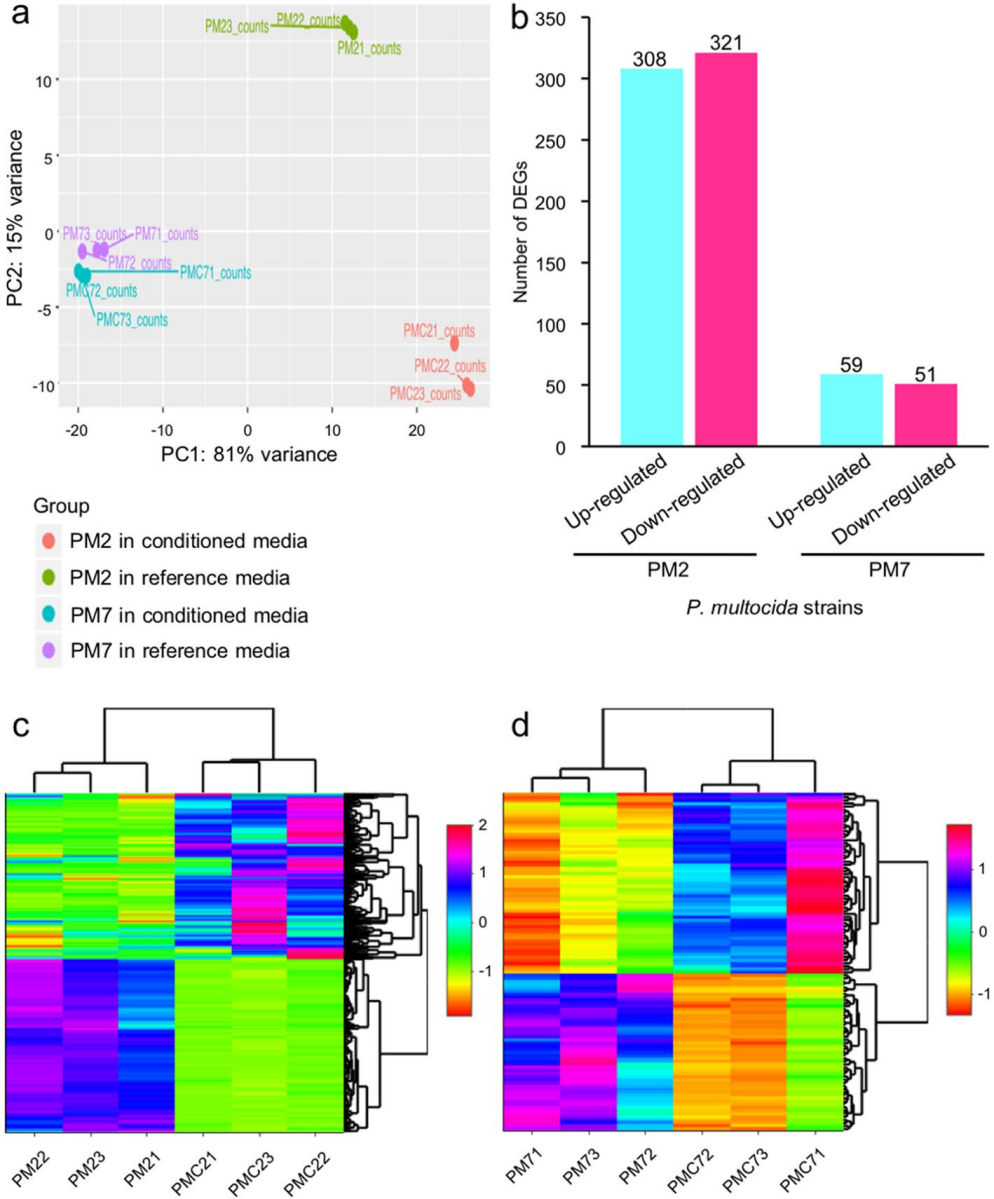
Transcriptomic analysis of *P. multocida* isolates PM2 and PM7 cultured in the complete media (BHIB) and conditioned media for 10 h. **A** Principal component analysis of the transcriptomes of these two *P. multocida* isolates grown in BHIB, and the *Aeromonas* conditioned media. **B** Comparison of the summative number of differentially expressed genes (DEGs) between the PM2 and PM7 isolates. Heatmaps represent the number of DEGs from the growth of PM2 (**C**) and PM7 (**D**) isolates in these two media conditions and among the three experimental replicates

From three replicates of each experimental condition (PM2 and PM7 isolates in BHIB and conditioned media), the total RNA concentration ranged between 659.40 to 1,134.10 ng/µL for the PM2 and 300.30 to 480.50 ng/µL for the PM7. The 260/280 ratios were between 1.86 and 2.01, and the 260/230 ratios were between 0.85 and 1.35. The NovaSeq Illumina sequencing platform with the 100-bp pair-ended library generated more than 20 million raw reads in all sample groups, and 185 million reads were assigned to 2,053 genes of *P. multocida*. Four datasets (two bacterial strains and two types of media with three replicates) of transcripts were analysed to identify differential gene expression. The PCA analysis showed distinct transcriptome profiles between the two *P. multocida* isolates and the media types (Fig. 1 A). Nearly all variance (96%) could be explained by the first two components (81% for PC1 and 15% for PC2). The transcriptomes of *P. multocida* isolate PM2 growth under the two media conditions were more distant than the isolate PM7 (Fig. 1 A).

### Analysis and clustering of differentially expressed genes

The read mapping process showed more reads from the growth in BHIB (17,000,629 reads for PM2 and 17,663,003 reads for PM7) were mapped to the reference than the growth in conditioned media (9,815,993 reads for PM2 and 17,118,502 reads for PM7). This mapping provided 2,053 genes: 1930 genes for PM2 and 2052 genes for PM7. The average number of reads mapped to each gene was higher for the growth in BHIB (8,807 reads for PM2 and 8,606 reads for PM7) than the growth in the conditioned media (5,088 reads for PM2 and 8,378 reads for PM7).

All 2,053 mapped genes were subjected to the differential gene expression analysis to contrast the two *P. multocida* growth conditions. Although the number of mapped genes (2,052 genes) was higher in the isolate PM7 than those of the PM2 isolate (1,930 genes), the number of differentially expressed genes (DEGs) was greatly lower in the PM7 isolate. The total number of 703 DEGs (log_2_ fold change value > |2| and adjusted *p*-value < 0.05) was obtained and annotated. The PM2 isolate had 629 DEGs which included 308 upregulated and 321 downregulated genes, whereas the PM7 isolate had 110 genes which contained 59 upregulated and 51 downregulated genes (Fig. 1B). These two *P. multocida* isolates shared the same 35 DEGs, including *acpT*, *algH*, *aroA*, *DUF5339 domain-containing gene*, *fumC*, *fxsA*, *grcA*, *grxA*, *hyaE, hemH*, *hisC*, *hrpA*, *kpsM*, *kpsE*, *lsrA*, *menI*, *mipA/ompV*, *MTHFS*, *murQ*, *murP*, *rpoB*, *rpoH*, *rseA*, *rbsD*, *rsuA*, *rraB*, *rpsH*, *rplY*, *rlmE*, *TC.PIT*, *YhcH/YjgK/YiaL family protein-coding genes*, *seqA*, *speF*, *wza/gfcE*, and *yccA* (Fig. 1B, 1C, and 1D). Three replicates of the PM2 growth in both BHIB (PM21, PM22, and PM23) and conditioned media (PMC21, PMC22, PMC23) showed the same global trend of gene expression, while more variation had been observed for the PM7 isolate.

The number of read changes was used to classify *P. multocida* gene expression responses into seven categories to depict an overview of this bacterial response, including strongly increased (11 genes for PM2 and 10 genes for PM7), moderately increased (7 genes for PM2 and 8 genes for PM7), mildly increased (145 genes for PM2 and 186 genes for PM7), not different (985 genes for PM2 and 1,417 genes for PM7), strongly decreased (24 genes for PM2 and 4 genes for PM7), moderately decreased (32 genes for PM2 and 10 genes for PM7), and mildly decreased (727 genes for PM2 and 418 genes for PM7) (Table 1). Less than 20 genes of both isolates were highly and moderately increased when growing in the conditioned media. Nearly 60 genes of the PM2 strongly and moderately declined, while less than 15 genes were observed in the PM7 isolate.

**Table 1:** Response levels of *Pasteurella multocida* growth under the conditioned media compared with the complete media, classified by the number of read changes

Response level	Number of read changes	Number of corresponding genes of *P. multocida*	
		PM2	PM7
Strongly increased	> 50,000 reads	11	10
Moderately increased	20,000–50,000 reads	7	8
Mildly increased	1,000–20,000 reads	145	186
Not different	0–1,000 reads	985	1417
Strongly decreased	> 50,000 reads	24	4
Moderately decreased	20,000–50,000 reads	32	10
Mildly decreased	1,000–20,000 reads	727	418

Depending on the grouping above, the strongly increased genes involved the genes coding for 11 proteins of the isolate PM2 (4-hydroxythreonine-4-phosphate dehydrogenase PdxA, 50 S ribosomal protein L20, 50 S ribosomal protein L35, ribose 5-phosphate isomerase B, sugar-binding transcriptional regulator, TRAP transporter substrate-binding protein, galactose/glucose ABC transporter substrate-binding protein MglB, dihydroxyacetone kinase subunit DhaK, dihydroxyacetone kinase subunit DhaL, sigma-E factor negative regulatory protein, and sucrose-specific PTS transporter subunit IIBC) and 5 proteins for the isolate PM7 (helix-turn-helix domain-containing protein, nitrate/trimethylamine N-oxide reductase NapE/TorE, alternative ribosome-rescue factor A, DUF5377 domain-containing protein, and hypothetical protein).

For the 28 strongly decreased genes in Table 1, 4 protein-coding genes (encoding for molecular chaperone DnaK, ATP-dependent chaperone ClpB, pyridoxal 5’-phosphate synthase lyase subunit PdxS, and porin OmpA) were highly decreased in both isolates. Twenty of these genes were strongly reduced in particularly the isolate PM2, including the synthesis of 23 S rRNA (uridine(2552)-2’-O)-methyltransferase RlmE, acetate kinase, ATP-dependent zinc metalloprotease FtsH, catabolite repressor/activator, chaperonin GroEL, class II fumarate hydratase, cytochrome ubiquinol oxidase subunit I, efflux RND transporter periplasmic adaptor subunit, efflux RND transporter permease subunit, endopeptidase La, formate C-acetyltransferase, formate transporter FocA, fumarate reductase (quinol) flavoprotein subunit, HslU-HslV peptidase ATPase subunit, oligopeptidase A, phosphoglycerate kinase, phosphopyruvate hydratase, pyridoxal 5’-phosphate synthase glutaminase subunit PdxT, pyruvate kinase, and thioredoxin-disulfide reductase.

From the moderately increased and decreased genes, a gene involved in synthesising RNA polymerase sigma factor RpoE showed an increased expression in the isolate PM2, but the opposite for the isolate PM7. Three moderately-expressed genes (encoded for cytochrome-C peroxidase, glutathione peroxidase, and porin) were decreased in both isolates, while the remaining genes showed opposite expression directions between these isolates. For the mildly responded genes, 5 genes relevant to aldolase, autoinducer-2 ABC transporter ATP-binding protein LsrA, autoinducer-2 ABC transporter permease LsrC, hydroxypyruvate isomerase family protein, and NAD(P)-dependent oxidoreductase showed slightly increased response in both isolates. In comparison, 290 genes showed a low decreased level of response in these isolates, i.e., genes encoded for peroxiredoxin C, PTS mannose/fructose/sorbose transporter subunit IIC, membrane protein FxsA, MgtC/SapB family protein, and OmpH family outer membrane protein.

### Comparative functional analysis of the differentially expressed genes

**Fig. 2:**
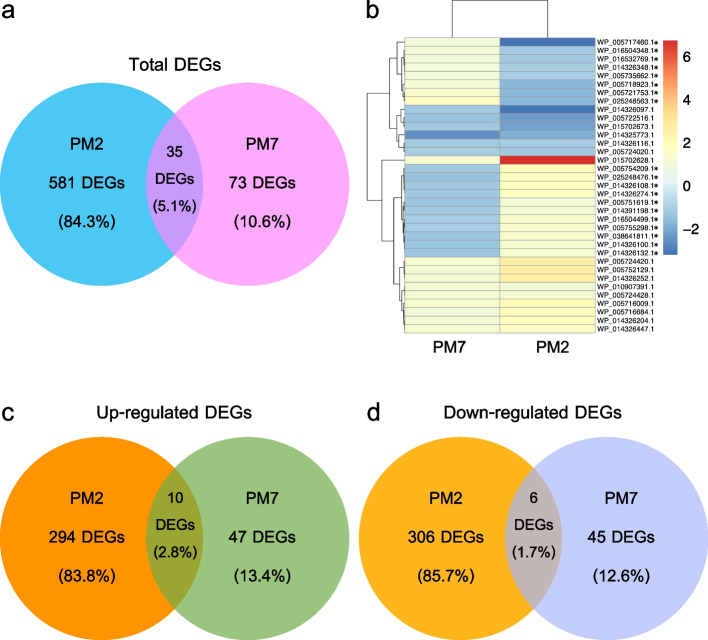
Numeric comparison of the differentially expressed genes from the growth of isolates PM2 and PM7 in the complete and conditioned media: (**A**) the total number of DEGs, (**B**) shared DEGs between the two isolates, (**C**) upregulated DEGs, and (**D**) downregulated DEGs. Asterisk represented the gene that had different responses when compared between the two isolates

To understand the gene expression between the two *P. multocida* isolates with different capsular serotypes, the compared transcriptome profiles of the PM2 and PM7 isolates grown in the complete and conditioned media revealed 35 shared DEGs, which were classified into five biochemical pathways, i.e., metabolism, genetic information processing, environmental information processing, cellular processes, and diseased pathways (Fig. 2A). Ten of these DEGs were significantly upregulated in both isolates, for example, *grxA*, *lsrA*, *murPQ*, *rbsD*, *rplY*, *rseA*, and *TC.PIT* (Fig. 2C). The function of these genes was related to ABC transporters of amino acids and nucleotides, quorum sensing, sugar metabolism, and genetic information processing. Six DEGs (*fumC*, *hyaE*, *minA/ompV*, *MTHFS*, *rpsH*, and *speF*) associated with metabolisms, signaling, and cellular processes were significantly downregulated in these two isolates (Fig. 2D). Another 19 DEGs showed different expression patterns between the PM2 and PM7 isolates, including *aroA*, *DUF5339 domain-containing protein encoding gene*, *fxsA*, *grcA*, *hemH*, *hisC*, *hrpA*, *kpsE*, *kpsM*, *LYS5*, *menI*, *rlmE*, *rpoB*, *rraB*, *rsuA*, *seqA*, *wza*, *yhcH*/*yjgK*/*yiaL family gene*, and *yccA* (Fig. 2B). There were 581 DEGs exclusively expressed in the PM2 isolate, while considerably less DEGs (73 genes) were uniquely observed in the PM7 isolate (Fig. 2A).

**Fig. 3:**
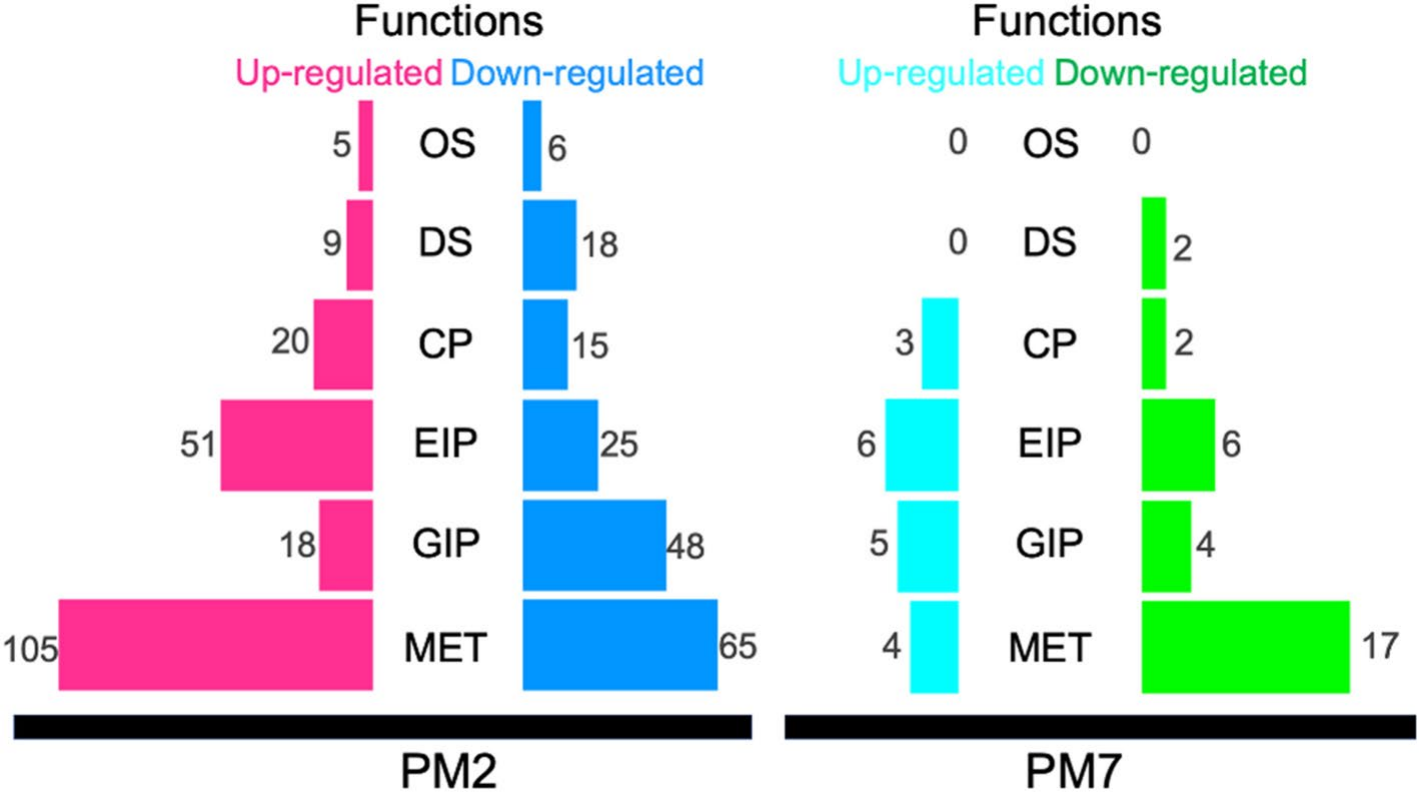
Number of functional ontologies assigned to the annotated differentially expressed genes (upregulated and downregulated genes) of *P. multocida* isolates PM2 and PM7 grown in the complete and conditioned media. Six significant categories included diseased pathway (HD), cellular process (CP), environment information processing (EIP), genetic information processing (GIP), metabolism (MET), and organismal system (OS)

The sets of upregulated and downregulated DEGs from the PM2 and PM7 isolates were functionally annotated and categorised into six primary functions: metabolism, genetic information processing, environmental information processing, cellular processes, organismal systems, and diseased function (Fig. 3). Three hundred and fourteen KEGG Ontologies (KOs) were annotated from the BlastKOALA program. There were 163 and 130 KOs annotated from the upregulated and downregulated DEGs of the PM2 isolate. In comparison, fewer number of KOs were obtained from the upregulated and downregulated DEGs (15 and 20 KOs) of the PM7 isolate. Among the six significant pathways, metabolisms were the primary function of DEGs in both *P. multocida* isolates. At the same time, environment and genetic information processing were the second- and third-ranked functions that displayed alteration under the growth in the conditioned media.

From this study’s 689 DEGs and 314 unique KOs, *P. multocida* isolate PM2 revealed 105 and 65 unique KOs related to the upregulated and downregulated DEGs of the metabolic pathways. Fewer KOs were determined in this isolate for the cluster of environmental information processing, genetic information processing, cellular process, organismal system, and disease pathways, respectively (Fig. 3). Compared with the PM7 isolate, a considerably lower number of DEGs and unique corresponding KOs were observed in five categories, except none for the organismal system (Fig. 3).

Considering the metabolic pathway cluster, 84 minor pathways were identified, including amino acid metabolism, biosynthesis of other secondary metabolites, carbohydrate metabolism, energy metabolism, glycan biosynthesis and metabolism, lipid metabolism, metabolism of cofactors and vitamins, metabolism of other amino acids, metabolism of terpenoids and polyketides, and nucleotide metabolic pathway. There were 167 DEGs associated with this metabolic pathway cluster: 148 genes only in the isolate PM2 and 11 genes exclusively in isolate PM7. Most of these genes (103 genes) were upregulated and associated with the biosynthesis of secondary metabolism and microbial metabolism in diverse environments. The metabolic genes related to metabolisms of carbohydrate, energy, amino acid, co-factors, and vitamins were significantly expressed in both PM2 and PM7 isolates, including *aroA*, *E4.2.1.2B*, *E4.1.1.17*, *hemH*, *hisC*, LYS5, MTHFS, *murQ*, *murP*, and *menI*. For the genes uniquely expressed in the PM2 isolate, the expression of *dhaKL* (glycerolipid metabolism) and *rpiB* (pentose, fructose, and mannose metabolism) appeared to be the highest. These genes were associated with the metabolic pathways, biosynthesis of secondary metabolites, microbial metabolism in a diverse environment, and carbon metabolism. The transcriptions of genes related to tyrosine metabolism (*hpaDFG*) were also expressed only in the isolate PM2. Most of the genes involved in carbohydrate and sugar metabolism (*FBA*, *galKLMT*, *lyaX*, *sucCD*, *srlA*, and *scrA*) were significantly upregulated only in the isolate PM2, but only *glpX* was found to be exclusively upregulated in the isolate PM7 for these functions. The down regulation of *E4.2.1.2B*, *E4.1.1.17*, and *MTHFS*, and the upregulation of *murQ* and *murP* were the same in both isolates. For the expression of the *mur* gene family, all *murACDEFGIPQ* genes were significantly upregulated in both isolates, except that the upregulation of *murP* and *Q* were observed in the isolate PM7. The expression direction of *aroA*, *hisC*, *hemH*, *LYS5*, and *menI* were opposite when compared between the two isolates.

For the environmental information processing cluster, membrane transport and signal transduction pathways were the major groups of significant DEGs (82 DEGs). The genes involved in the iron (III) transport system, ribose transport system, and two-component system (*afuABC*, *dctPQ*, *rbsABC*, *tcaAB*, and *uhpAB*) were significantly upregulated in the isolate PM2 while the expression of *kpsEM*, *modAB*, and *macB* in these functional clusters was significantly downregulated in the isolate PM7. Comparing DEGs functioned in these pathways, *lsrABCD* showed the highest level of expression, and these genes were autoinducers and ABC transporters in the bacterial communication and community. The *afuBC* encoded the iron (III) transporters, and they were upregulated only in isolate PM2, while *afuA* was expressed in both PM2 and PM7. Similarly, the capsular polysaccharide transporter genes (*kpsEMT*) were expressed in both isolates, and all these genes were upregulated, except the down regulation of *kpsEM* in the isolate PM7. Twelve genes associated with the two-component regulatory system were upregulated in the isolate PM2, including *csrA*, *dnaA*, *dcuB*, *dctMPQ*, *pgtC*, *qseB*, *ttrS*, *torD*, *tctAB*, *uhpABT*, and *wza*. In contrast, only one gene (*phoR*) was significantly upregulated in the isolate PM7.

The genes involved in transcription, translation, DNA replication and repair, protein folding, sorting, and degradation were significantly expressed in *P. multocida.* Three gene families (*rplCDEKNOPTUXY*, *rpmABEFGHIJ*, and *rpsAFHJMNORTU*) involved in ribosomal subunit proteins were significantly expressed in the isolate PM2, while *rplY* and *rpsH* were shown in the other isolate. The expression of most genes (23 genes) significantly declined except the upregulation of *rplOPTY*, *rpmFIJ*, and *rpsM*. The genes involved in aminoacyl-tRNA biosynthesis were expressed considerably only in the isolate PM2, including *aspS*, *CARS*, *FARSAB*, *NARS*, *QARS*, *KARS*, and *SARS*, same as those related to protein export (*secA*, *ftsY*, and *yajC*), and DNA mismatch repair (*scbB*, *dnaE*, *holC*, *xseB*, and *recJ*).

Four pathways involved in cellular processes, including transport and catabolism, cell growth and death, cell motility, and cellular community, were significantly expressed in *P. multocida*. The expression of quorum sensing genes was raised considerably in both isolates. The presence of *lsrABCDFGKR* was shown in the isolate PM2, while only the expression of *lsrA* was observed in the other isolate. The significant expression of biofilm formation genes (*csrA*, *glgC*, *lsrR*, and *wza*) was increased only in the isolate PM2. In contrast, *arcA*, *crr*, *fis*, *gcvA*, *oxyR*, and *wza* were shown to be significantly downregulated in both isolates. The genes involved in apoptosis (*ahpC*) and necroptosis (*htpG*) were exclusively downregulated in the isolate PM2, while no significant expression was observed in the other isolate.

Another major pathway included 11 genes (*dgkA*, *dnaK*, *E.1.1.1.40*, *FBP*, *galT*, *groEL*, *GST*, *htpG*, *katE*, *PGAM*, and *PK*) associated with organismal system pathways which were significantly expressed only in the isolate PM2. These genes involved ageing, immune system, and environmental adaptation.

The diseased pathway was the last significant pathway significantly expressed in *P. multocida* under the coculture in the conditioned media. The genes involved in infectious disease were significantly downregulated, including *fhaC* and *gapA*, which involved in hemolysin activation or protein secretion. The two *P. multocida* isolates revealed the significant downregulation of the genes involved in beta-lactam resistance, including *acrA*, *acrR*, *mrcA*, and *oppA* in the isolate PM2 and *ampG* in the other isolate, similar to those involved in cationic antimicrobial peptide (CAMP) resistance (*acrA* and *sapABCD*) in the isolate PM2.

## Discussion

*P. multocida* is an opportunistic/pathogenic bacterium in the porcine respiratory tract colonised by several other normal flora and occasionally pathogens [28]. Our study focused on the porcine isolates of *P. multocida* harbouring two capsular serotypes (A and D) cultured in the conditioned media of *A. caviae* previously isolated from porcine respiratory tracts. The previous study examined the growth of these *P. multocida* isolates in the conditioned media prepared by several respiratory bacteria and revealed that the media prepared from *A. caviae* effectively inhibited the growth of *P. multocida* [28]. Hence, these current transcriptomic studies paved the aim to understand the interaction between *A. caviae* and *P. multocida*. Firstly, the PCA analysis of the RNAseq data suggested different gene expression responses during the growth of *P. multocida* isolates in the complete and the conditioned media, as well as the difference in responses between the two isolates. *P. multocida* with capsular serotype D (PM2) showed a higher ability to grow and survive better than serotype A (PM7) in both media types. This was supported by considerably more mapped reads from the PM2 isolate in response to the co-culture in the conditioned media. The response of different *P. multocida* strains also corresponded with the previous study of Cheng et al. that compared the expression of virulent genes in *P. multocida* isolates and found that the high-virulence strains displayed a higher level of cellular proliferation and increased expression of seven virulence genes compared to the low virulent strain [40]. This could also imply that the isolate PM2 would be much adaptable to the existing unfavored environments generated by the typical flora community in the tract, whereas the PM7 might be easier controlled.

Comparing the transcriptomes of two *P. multocida* isolates, this study identified a different pattern of the DEGs among these two isolates of capsular types A and D in response to the conditioned media. Many of the shared DEGs had metabolic functions. The upregulation of genes in the pentose phosphate pathways suggested that *P. multocida* had to adjust its metabolic pathways during the growth in conditioned media to earn necessary nutrients (sugars and amino acids) for survival. This pentose phosphate pathway is the main path of carbohydrate oxidation in *P. multocida*, involving the growth and resting stages of the bacterial cells [44]. The *dhaKL* genes were part of the phosphotransferase system (PTS), which controlled carbohydrate uptake and carbon metabolism. The phosphotransferase system is one of the significant sugar uptake in bacteria, involving sugar transport and phosphorylation [45]. The highest expression level of these genes in the isolate PM2 suggested that survival and better growth of this isolate in the nutrient-scarce conditioned media were achieved by increasing the carbohydrate and carbon source uptakes. Conversely, the isolate PM7 downregulated most of these genes, presumably leading to its slower propagation in the conditioned media.

For the thrive of *P. multocida* in the conditioned media, the expression of genes in the environmental information processing function provided essential signals for the bacteria to adjust their metabolisms accordingly. The highly expressed genes (*afuABC* and *lsrABCD*) involved in the synthesis of ATP binding proteins of the isolate PM2 suggested the requirement of ATP in the ATP-dependent reactions necessary for the nutrient acquisition and survival of this isolate in the *Aeromonas* conditioned media, including the upregulation of iron (III) transport system, ribose transport system, and methyl-galactoside transport system which relied on the drive of the ABC (ATP-binding cassette) transporters. Iron is the essential nutrient that plays a vital role in the electron transport chain and several metabolic pathways [46], as well as the pathogenesis and survival of *P. multocida* [47, 48]. For the case of this study, the ability of the isolate PM2 to sequester the limited availability of irons helped it grow better in the conditioned media compared to the isolate PM7. This could be supported by the high expression level of the *afuABC* genes in the isolate PM2. Moreover, He et al. [47] showed that the expression of iron-related genes, including *afuC* in *P. multocida*, influenced differential virulence of two *P. multocida* strains. These results would also suggest the higher virulence potency of the isolate PM2.

In the conditioned media, when *P. multocida* used all glucose, the bacteria consumed other sugar sources, such as ribose sugars which were the preferred energy source and were important to DNA and RNA metabolisms [49]. The ribose transporters genes *lsrABC* were also involved in the bacterial communication by the secretion of chemical signaling molecules [50, 51]. The increased expression of these genes highlighted the possible response of *P. multocida* to certain chemicals released into the conditioned media by *A. caviae* during the media preparation. These conditioned media were obtained by filtering the spent BHIB after the growth of *A. caviae*. The quorum sensing process is one of the pathways that bacteria use for communication to share environmental and population density information [52]. *P. multocida*, *Actinobacillus pleuropneumoniae*, and other members of the *Pasteurellaceae* family produced AI-2-like molecules, which were the quorum-sensing autoinducers [53]. The bioluminescence study of Molott and Lo detected the autoinducer molecules in the bacterial culture supernatant, indicating these molecules in the quorum sensing mechanism regulate the gene expression under specific conditions [53]. Many Gram-negative bacteria also encoded this gene, e.g., AI-2-like molecules of *E. coli* used in the quorum sensing-mediated control of this bacterial virulence [54]. Therefore, the isolated PM2 could employ these sensing pathways to monitor the unsuitable condition and alter its gene expression to overcome this pressure.

To adapt and respond to the conditioned media from *A. caviae*, the down regulation of genes related to the genetic information processing function could be the strategy to lower unnecessary mechanisms, including the protein synthesis of unurgent proteins and focus on the crucial pathways for survival [55]. This could be clearly shown by the suppressed expression of the ribosomal proteins constituting small and large subunits of the ribosome. These results implicated *P. multocida* adaptation under the growth in the conditioned media by enhancing the crucial energy uptake and nutrient-relevant pathways and slowing down the unurgent or high energy-consuming processes, interestingly including genes involved in drug resistance (beta-lactam and cationic antimicrobial peptides or CAMPs).

## Conclusion

Our transcriptomic results successfully described possible molecular mechanisms occurring during the growth of *P. multocida* isolates in the *A. caviae* conditioned media, which included the altered expression of genes involved in metabolisms, environmental and genetic information processing, cellular processes, and diseases pathways. The high expression level of genes belonging to the energy and nutrient metabolisms were keys to the growth and adaptation to the conditioned media, together with the decreased expression of those in the unurgent pathways, including translation and antibacterial resistance. This transcriptomic analysis also displayed the distinct capability of the two isolates of *P. multocida* and the preference of the capsular type D isolate in response to the tough environment of the *A. caviae* conditioned media. Therefore, controlling the environmental sensing and nutrient acquisition mechanisms of *P. multocida* would possibly prevent overpopulation of this bacteria and reduce the chance of becoming an opportunistic pathogen.

## Materials and methods

### Bacterial strains and growth conditions

Two porcine isolates PM7 (serotype A) and PM2 (serotype D) of *Pasteurella multocida* and one *Aeromonas caviae* isolate NS21 from the bacterial collection previously isolated from porcine respiratory tracts [28] were revived and grown in the tryptose blood agar (Oxoid, United Kingdom) containing 5% cow blood and incubated at 37^o^C for 24 h before use.

### Preparation of complete and conditioned media

Brain heart infusion broth (BHIB; TM MEDIA, Titan Biotech Ltd, India) was prepared as described by the manufacturer and used in the conditioned media preparation as previously described by Hanchanachai et al. [28]. Briefly, a single colony of *A. caviae* strain NS21 was sub-cultured into 40 mL BHIB and incubated at 37^o^C, 180 rpm for 48 h. The bacterial cells were collected by centrifuging at 4,800 *x g* at room temperature for 15 min. The supernatant was filtered by a 0.2 μm polyethersulfone (PES) membrane filter (Whatman, United Kingdom) using a 50 mL syringe (Nipro, United States). As the *A. caviae* conditioned media completely inhibited the growth of *P. multocida*, the dilution of the media was required to allow enough cell pellets for the RNA extraction. Two inhibitory concentrations (IC_20_ and IC_50_) of the *A. caviae* conditioned media were determined for these *P. multocida* isolates by growing them in 10% and 20% v/v conditioned media at 37^o^C for 10 h. The IC_20_ and IC_50_ values were calculated using the linear regression equations from the growth curve generated by the average OD_600_ values of the triplicate cultures. The optimal IC value for each isolate’s growth was chosen for the conditioned media preparation and the following steps.

### Co-culture of *P. multocida* isolates in the *A. caviae* conditioned medium

This study was organised into four experimental groups of two *P. multocida* isolates (PM2 and PM7), and two types of media (complete medium BHIB and conditioned medium prepared by *A. caviae*), and each group was conducted in triplicate. A single colony of these two *P. multocida* isolates was inoculated in the control media and incubated at 37^o^C for 10 h. Then 40 µl of the bacterial culture was transferred into 40 mL of BHIB, and the *A. caviae* conditioned medium (at the optimal IC values) before incubating at 37^o^C, 180 rpm for 10 h and harvesting when the absorbance at 600 nm was more than 1.0.

### Bacterial RNA isolation, library construction, and next-generation sequencing

After cultures, the bacterial cells were harvested by centrifugation at 13,500 rpm for 5 min at 4^o^C. RNAs were isolated using the TRIzol reagent (TRIGEN/MRC, Cincinnati, United States) as described by the manufacturer. Briefly, 1 mL of MRC Tri-Reagent® was added to the bacterial pellet and homogenised using pipetting. Then, 200 µL of chloroform were added to the mixture and vortexed vigorously before incubating on ice for 3 min. The mixture was centrifuged at 12,000 *x g* at 4˚C for 15 minutes to separate into a lower red phenol-chloroform, interphase, and a colourless upper aqueous phase. 500 µL of the upper aqueous phase containing RNA were transferred to a new tube, and the RNAs were isolated by adding 500 µL of isopropanol into the aqueous phase and incubated at -80˚C for 10 min before centrifugation at 12,000 *x g* at 4˚C for 10 min and discarding the supernatant. The RNA pellets were resuspended in 1 mL of 75% ethanol and centrifuged at 7,500 *x g* at 4˚C for 10 min. The remaining supernatants were discarded by pipetting, and the RNAs pellets were air-dried for 10 min before resuspending in 85 µL of RNase-free water. The samples were stored at -80˚C until use. All manipulations were carried out on the ice or at 4^o^C. The RNA concentration and quality were determined by measuring absorbance at 260 and 280 nm and visualising by 1% agarose gel electrophoresis. These total RNA samples from two bacterial isolates, two types of media, and three experimental replicates were sequenced using the NovaSeq Illumina platform and 100-bp paired-end libraries (Macrogen, Korea). The 100 paired-end libraries were prepared from the extracted RNAs using random fragmentation of the cDNA samples, followed by 5’ and 3’ adapter ligation. The qPCR was used to quantify the prepared libraries according to the Illumine qPCR quantification protocol guide.

### RNA sequencing data analysis

Quality assessment of the sequence reads (e.g., the quality per base and adapter content) was evaluated with the FastQC program v0.11.5 [56]. The reads were mapped to the reference genome of *P. multocida* strain NCTC10322 (accession number NZ_LT906458) using the bwa program version 0.7.17.4 [57] with default parameter setting to pair-end mode and the bwa-MEMs algorithm [57]. The mapped contigs were annotated using the reference annotation file (GCF_900187275.1_51765_F01_genomic.gtf) of the reference genome (accession number GCF_900187275.1) from the NCBI database. The annotation process was done using the best-placed reference protein set and the GeneMarkS-2 + method in the NCBI prokaryotic Genome Annotation Pipeline (PGAP) [58, 59]. The mapped reads were counted using the samtools idxstats program version 1.10 [60], and the counted results were used in differential gene expression analysis and clustering of bacterial responsive levels.

### Differential expression analysis

The DESeq2 program (Galaxy version 2.11.40.6 + galaxy1) [61] on the Galaxy server [62, 63] was used to analyse the mapped read data of *P. multocida* and identify differential gene expression (DEG). The gene expression was compared between different experimental conditions, including conditioned and complete media, and the two isolates of *P. multocida* (PM2 and PM7). The differentially expressed genes were extracted based on the absolute fold-change values (FC) greater than two and the Benjamini-Hochberg adjusted *p*-values smaller than 0.05. The Z-scores of the differentially expressed genes were computed from the normalised counts of the DESeq2 analysis, and the heatmap was used to visualise different expression of these genes. These differentially expressed genes were annotated and functionally classified using the BlastKOALA program version 2.2 [64]. The genes and their functions were compared in terms of number and expression level using gene code and their log_2_ fold change values.

### Statistical analysis of the transcriptomic data

The comparison of differential gene expression was tested at the statistical significance level of *p* < 0.05. To determine differentially expressed features from count tables of the transcriptome results, the normalised mean counts were averaged over all samples from all conditions using DESeq2’s median of ratios method [65], and the standard error was estimated from the log2 fold change values. The Wald test adjusted the p-value before the comparison by the Benjamini-Hochberg procedure, which controls the false discovery rate using the DESeq2 program.

## Data Availability

The data was submitted to the NCBI GEO database with the accession number GSE211697.
